# Warming effects on the life cycles of two parasitic copepods with different invasion histories

**DOI:** 10.1002/ece3.11485

**Published:** 2024-06-25

**Authors:** Elli Rosa Emilia Jolma, Ana Born‐Torrijos, Hans Heesterbeek, Anieke van Leeuwen, Sonja Maria van Leeuwen, Robert H. Twijnstra, K. Mathias Wegner, David W. Thieltges

**Affiliations:** ^1^ Department of Coastal Systems NIOZ Royal Netherlands Institute for Sea Research Den Burg, Texel The Netherlands; ^2^ Department of Population Health Sciences Utrecht University Utrecht The Netherlands; ^3^ Alfred Wegener Institute – Helmholtz Centre for Polar and Marine Research, Coastal Ecology, Waddensea Station Sylt, List Sylt Germany; ^4^ Groningen Institute for Evolutionary Life‐Sciences (GELIFES) University of Groningen Groningen The Netherlands

**Keywords:** climate change, copepod, invasive species, *Mytilicola intestinalis*, *Mytilicola orientalis*, *Mytilus edulis*, parasite

## Abstract

Climate change may exacerbate the impact of invasive parasites from warmer climates through pre‐existing temperature adaptations. We investigated temperature impacts on two closely related marine parasitic copepod species that share the blue mussel (*Mytilus edulis*) as host: *Mytilicola orientalis* has invaded the system from a warmer climate <20 years ago, whereas its established congener *Mytilicola intestinalis* has had >90 years to adapt. In laboratory experiments with temperatures 10–26°C, covering current and future temperatures as well as heat waves, the development of both life cycle stages of both species accelerated with increasing temperature. In the parasitic stages, the growth of the established invader increased evenly from 10°C to 22°C, whereas the recent invader barely grew at all at 10°C and grew faster already at 18°C. In contrast, temperature had little effect on the transition success between life cycle stages. However, the highest temperature (26°C) limited the egg development success of the established invader and the host entry success of both species, whereas the infection success of the established invader increased at 18°C and 22°C. In general, our experiments indicate that the main effect of temperature on both species is through development speed and not life cycle stage transition success. Based on regional long‐term temperature data and predictions, the numbers of completed life cycles per year will increase for both parasites. The established invader seems better adapted for low current temperatures (around 10°C), whereas the more recent invader barely develops at these temperatures but can cope in high temperatures (around 26°C). Hence, pre‐existing temperature adaptations of the recent invader may allow the species to better cope with heat waves.

## Graphical abstract

Warming temperatures may benefit invasive parasites that originate from warmer climates. We found that warming does enable faster parasite life cycles and a recent invader from a warm climate tolerated heat wave temperatures better than an established congener.

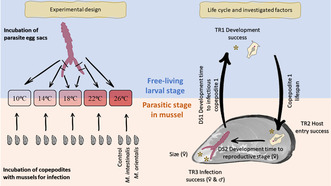

## 1 INTRODUCTION

Climate change and biological invasions are changing ecosystems at an unprecedented rate. However, their combined effects are only rarely investigated, although shifts beyond the environmental optimum of native species can favour the establishment and spread of invasive species (Ricciardi et al., 2013; Thuiller, 2007). Biological invasions can include micro‐ and macroparasites that can have profound effects on recipient ecosystems if they spill over to native species and cause emerging diseases, mediate competition between native and invasive hosts, or compete with native parasites (Dunn & Hatcher, 2015; Thieltges & Goedknegt, 2023). The thermal adaptations of invasive parasites can differ from those already inhabiting recipient ecosystems, and that may affect their competitive success in ecosystems undergoing rapid temperature increase due to climate change.

Temperature can directly affect parasites in two ways. On the one hand, it can affect the transition of parasites from one life cycle stage to the next. For example, in some trematode parasites rising temperatures increase the infectivity by an elevation in their activity levels (Barber et al., 2016), while the susceptibility of hosts can increase due to a comprised immune system function induced by heat stress (Karaer et al., 2023). On the other hand, temperature can affect parasites by speeding up their life cycles. Within the thermal range of ectothermic animals, an increase in temperature generally speeds up life cycles by increasing the metabolic rate and development speed in the absence of food limitation (Gillooly et al., 2001). The flipside of these sped‐up life cycles usually is that also the lifespan is shorter and adult size is often smaller at higher temperatures (Atkinson, 1994; Gillooly et al., 2001). For example, in trematode parasites an increase in temperature can cause high numbers of infective stages to be released from first intermediate hosts in short periods of time, resulting in high infection loads in the second intermediate hosts (Mouritsen & Jensen, 1997; Poulin, 2006). However, increasing temperature also typically makes free‐living infective stages consume their energy reserves faster resulting in higher mortality, which can lead to lower infection levels in second intermediate hosts of trematodes at high temperatures (Barber et al., 2016). Parasites are ideal candidates to investigate both types of consequences of temperature because the transitions between life cycle stages are very clearly defined, e.g., the transition from free‐living to parasitic life cycle stages during transmission. Furthermore, when parasites are invasive species originating from warmer climates, the adaptive potential of life cycle evolution can be investigated. Climate change may give recent invaders coming from warmer regions a competitive advantage over established invasive and native parasites due to pre‐existing temperature adaptations because of higher temperatures in their original range (Hellmann et al., 2008; Thuiller, 2007). In addition, they may have a larger capacity for adaptation to broader temperature ranges which is often seen in invasive species (Hellmann et al., 2008).

**FIGURE 1:**
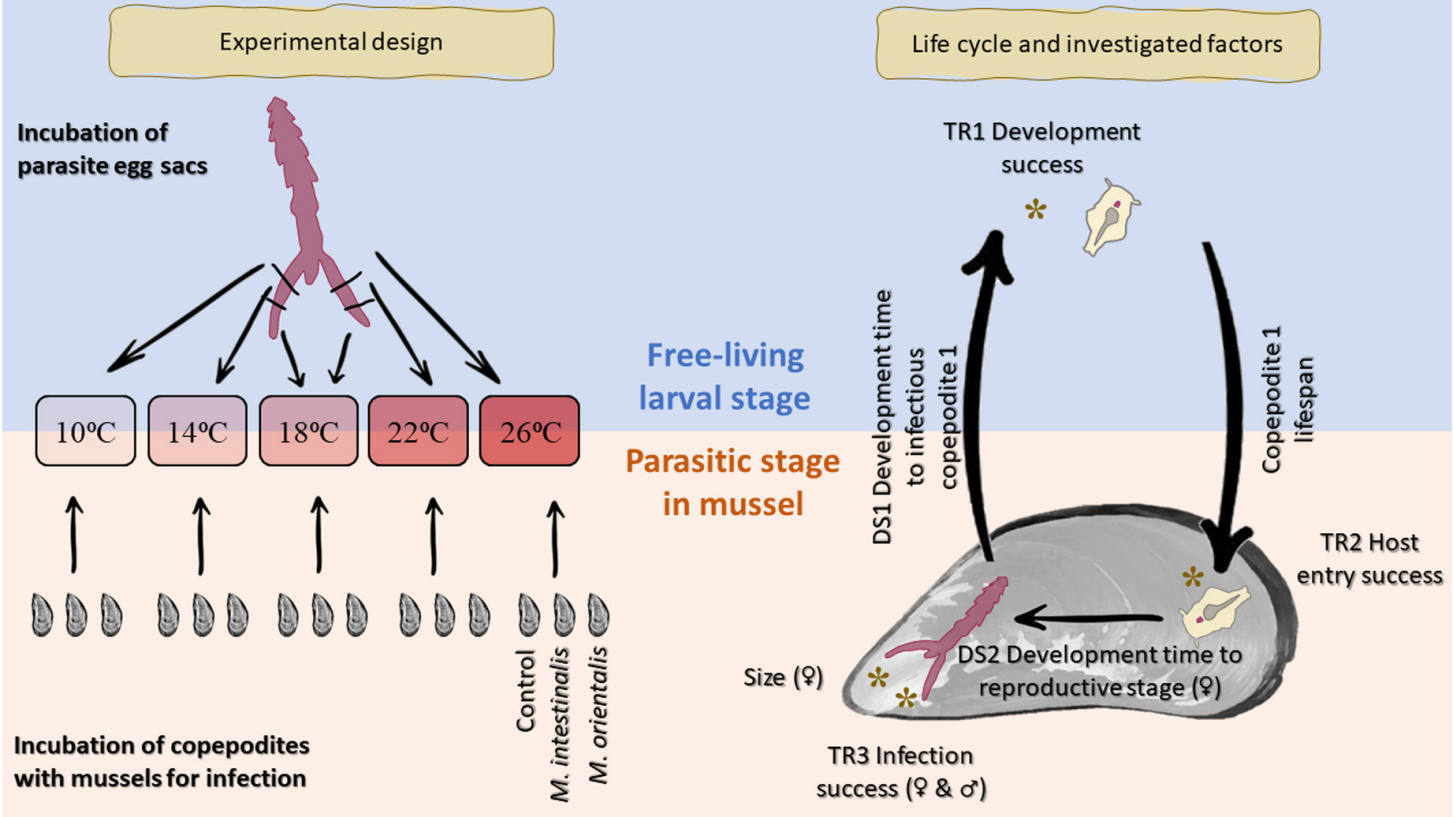
Temperature effects on life cycle stages of two closely related parasite species, *Mytilicola intestinali*s and *Mytilicola orientalis*, both infecting blue mussels. In the experiments, life cycle stages were evaluated at five different temperatures to estimate the impact of temperature on crucial life cycle transitions and duration of stages. On the right, asterisks represent measures used for estimating the success of life cycle transitions (TR1–TR3) and the size of egg‐bearing female parasites, arrows represent development times (DS1–DS2).

Here, we investigate temperature effects on two species of parasitic copepods that share the blue mussel (*Mytilus edulis*) as a host in their invaded range of the European Wadden Sea, a shallow temperate coastal ecosystem that is affected not only by the frequent arrival of invasive species but also by rapid sea water warming (Reise et al., 2023; Reise & van Beusekom, 2008; Royal Netherlands Institute for Sea Research, 2023; van Aken, 2008). Both parasite species have a simple life cycle with a short non‐feeding free‐living planktonic larval stage and the adult parasite developing inside the gastrointestinal tract of a bivalve (Gee & Davey, 1986; Goedknegt, Thieltges, et al., 2018; Figure 1). The two parasite species are closely related but have different invasion histories (Feis et al., 2019; Goedknegt, Thieltges, et al., 2018). The original geographical range of *Mytilicola intestinalis* is not known but the species was first described from the Mediterranean Sea in 1902 and arrived in the Wadden Sea already in the 1930s (Feis et al., 2019). While high mussel mortalities were observed during the initial phase of the invasion, infections are now more benign, and after approximately 90 years of presence this species can be considered as naturalised. The second species, *Mytilicola orientalis*, arrived in the Wadden Sea in the first decade of the 21st century, together with its principal host, the invasive Pacific oyster (*Magallana* (*Crassostrea*) *gigas*; Feis et al., 2019). In the Wadden Sea, *M. orientalis* has spilled over to several native bivalve species, including blue mussels and common cockles (*Cerastoderma edule*), whereas *M. intestinalis* has a more limited host range and does not infect Pacific oysters in the field (Goedknegt, Thieltges, et al., 2018) or in controlled infection experiments (Feis et al., 2022). The native range of *M. orientalis* is in the Western Pacific Ocean around southern Japan (Feis et al., 2019), where both winter (>13°C) and summer (>25°C) water temperatures are much warmer than in the Wadden Sea, where before climate change the year average water temperature was 10.5°C and temperatures went rarely >18°C (Japan Meteorological Agency, 2023; Oost et al., 2017; Royal Netherlands Institute for Sea Research, 2023). This may give this recent invader an adaptive advantage with increasing seawater temperatures through climate change.

We used an experimental laboratory approach to compare the temperature responses of the two parasite species with possibly different temperature adaptations based on their origin and the different time scales they have had for adapting to the new environment. With this, we hoped to learn whether recent invasive parasites from warmer waters could benefit from predicted future water temperature increase, compared to long‐established invaders that may act as a proxy for native parasites. In controlled laboratory infection experiments, we (i) determined the impact of temperature on the transition of parasites from one life cycle stage to the next and (ii) quantified temperature effects on the developmental rates throughout the life cycle. From the respective life cycle stage transition probabilities and the durations of life cycle stages under experimental conditions, (iii) we then estimated how the increase in regional water temperature from 1982 to 2021 and the predicted increase by 2100 will affect the number and timing of annual life cycles of both parasites to gauge their future impact on native hosts and ecosystems. We hypothesised that the predicted increase in water temperature would have a positive effect on the developmental rates and life cycle transition success of both species, and that the pre‐existing temperature adaptations of the recent invader would give it an advantage over its established and naturalised congeners.

## 2 METHODS

### 2.1 Temperature levels

The sea water temperature in the Wadden Sea is predicted to increase by 1–5°C from the baseline before climate change effects (1880–1990) by the year 2100, which means water temperature is getting closer to that in the native range of *M. orientalis* (Japan Meteorological Agency, 2023; Oost et al., 2017; Royal Netherlands Institute for Sea Research, 2023). Based on this, we chose a range of five temperatures (10°C, 14°C, 18°C, 22°C and 26°C) that reflect both the current and predicted temperatures of the Wadden Sea (Figure 1). 10°C and 14°C were chosen as approximate past and future mean annual water temperatures, and 18°C and 22°C as past and future mean temperatures for the warmest summer months (Reise & van Beusekom, 2008; Royal Netherlands Institute for Sea Research, 2023). On top of these four temperatures, we decided to add 26°C as a fifth temperature simulating future heat wave conditions (Figure 1). The effects of temperature and its interactions with parasite species were statistically estimated using temperature as a categorical variable, because we expected possible effects to be linear only in the Arrhenius range of temperatures up to peak performance that each parasite species is adapted to (Molnár et al., 2017). The large range of temperatures made it likely that some were outside that adapted range, and we aimed to assess where break points occurred in the temperature range, rather than to assess if there was a temperature effect in general.

### 2.2 Parasite life cycle

Both *M. intestinalis* and *M. orientalis* have simple life cycles with a short free‐living planktonic larval stage and a parasitic stage in the intestine of a bivalve (Figure 1). To understand the impact of temperature on these parasites, we investigated how it affects (1) their capacity to transition from one life cycle stage to the next (TR = transition success) and (2) the duration of completing the both life cycle stages (DS = development speed). For these aims, we used three measures to estimate transition success: (TR1) development success (of eggs), (TR2) host entry success (the capacity of free‐living stages to enter hosts) and (TR3) infection success (the capacity of free‐living stages to infect mussels) and we estimated the duration to complete both life cycle stages: (DS1) development time to infectious copepodite 1 and (DS2) development time from infectious copepodite to reproductive stage (Figure 1).

### 2.3 Free‐living larval stages

#### 2.3.1 Sources

Blue mussels were collected from intertidal mixed mussel‐oyster reefs in the southern Wadden Sea adjacent to the island of Texel on September 13, 2022, on April 25, 2023 and on May 9, 2023. The experiment included two seasons (autumn and spring) to cover a range of egg development temperatures and because of seasonal limitations in egg availability. Mussel intestinal tracts were dissected and female copepods with egg sacs were collected under a stereo microscope with 10× magnification (ZEISS SteREO Discovery V8). The species identity of each female was determined based on morphological features (Goedknegt, Thieltges, et al., 2018) and only females that had all morphological features consistent with those of one species were chosen for egg collection. 24 egg sacs were collected during each season resulting in a total number of 48. The ripeness of egg sacs was determined in the beginning of the experiment based on the presence of embryonic eyes (red dots).

#### 2.3.2 Egg incubation, development success, copepodite development and survival

Each pair of egg sacs was cut into five approximately similar‐sized egg sac sections with a scalpel and the sections were incubated in 12‐well cell culture plates at five different temperatures (48 × 5 resulted in 240 egg sac sections, Figure 1). This resulted in 20 cell culture plates in total (two plates per temperature per season = 24 replicates with 120 egg sac sections per season). The lids of the cell culture plates were insulated with radiator foil and each well contained 4 mL of filtered sterile sea water. The plates were checked under a stereo microscope with 10× magnification daily for the first 3 weeks and then daily on weekdays only. The dates of the following responses were recorded for each well in each plate: first hatching, first copepodite 1 (the infectious stage) and 50% mortality of all hatched parasites (see Gee & Davey, 1986 for a visual description of the life cycle stages). First hatching was recorded to evaluate consistency across development times while our main outcome of interest was developing all the way from eggs to the infectious stages (DS1 and TR1 in Figure 1) including two earlier non‐infectious stages (nauplius and metanauplius, Gee & Davey, 1986). The time of 50% mortality was recorded to enable estimating infectious lifespan of the copepodite 1 stage (time from reaching the infectious stage to 50% mortality).

### 2.4 Stages inside mussels

#### 2.4.1 Infection of mussels

Mussels (150 individuals) with a shell length (maximum anteroposterior axis) of 35–40 mm were collected from the North Sea at Sint Maartenszee (52.791559, 4.669481) on July 18, 2022, a location on the mainland close to Texel known to be free of *Mytilicola* spp. and trematode infections (0% infection prevalence of both groups was confirmed during summer–autumn of 2022 based on 100 mussel dissections). All epibionts were carefully removed and the mussels were divided up between the five experimental temperatures (Figure 1). The mussels were acclimated for the temperatures and laboratory conditions for at least 4 weeks and the treatment batches were started between August 18 and September 4, 2022 (for practical reasons only one batch was started daily). Within each of the five temperature treatments, the mussels were split into three infection treatments (control, *M. intestinalis*, *M. orientalis*), resulting in 15 different treatment combinations (Figure 1). Each batch of 15 mussels was started and handled together until the end of the experiment to minimise bias from any non‐treatment effects. Each batch was replicated 10 times (i.e., 10 treatment replications and 150 mussels in total).

The mussels were infected with infectious copepodite 1 stage larvae that originated from eggs sourced as described above for the preparation of the free‐living stages and incubated at room temperature (approximately 20–23°C). For infection with either *M. intestinalis* or *M. orientalis*, 24 infectious copepodites were added to a single mussel and incubated for 24–27 h in a 125 mL glass jar with approximately 1,000,000 cells of phytoplankton concentrate (Microalgae Mix, Acuinuga, La Coruña, Spain) and 100 mL of filtered sea water. Individual mussels of all temperature treatments of a batch were experimentally infected with copepodites originating from the same 2–3 parasite egg sacs from each parasite species to balance out the genetic and quality effects across temperature treatments. Control mussels received the same treatment but without infectious parasite stages. After the incubation, the mussels were rinsed, their length was measured to the closest 0.1 mm, and they were moved into individual plastic Kautex™ 200 mL jars. The 100 mL of water used for infection was filtered through a 4 μm filter and the number of infectious stages that were not taken up by the mussels was recorded under a stereo microscope to estimate the host entry success. The mussels were randomly placed in temperature‐controlled water baths with a flow‐through system that dripped 567 mL of filtered sea water into each jar per 24 h. 40,000,000 cells of phytoplankton concentrate (Microalgae Mix, Acuinuga, A Coruña, Spain) per mussel per 24 h were mixed into the incoming water (Figure S1). The photoperiod was set for 12 h of light and 12 h of dark. The system was cleaned every 14 days to prevent algal and bacterial build‐up.

#### 2.4.2 Dissection

Starting from 8 weeks after infection, two full batches (2 × 15 mussels, Figure 1) were dissected every 3 weeks, resulting in five time points: 8, 11, 14, 17 and 20 weeks. Mussel intestinal tracts were dissected and examined under a stereo microscope and the number of parasitic copepods was recorded. All parasites were photographed with 10× magnification (AxioCam ICc 3 or Pixel2 [with Android 11]) and the length of each parasite was measured (AxioVision LE [© Carl Zeiss MicroImaging GmbH] or Measure Pixels 1.0.0.0 [© Leisn 2021]). A subset was photographed and measured with both methods indicating that the difference between methods was 1%–8%. Damaged parasites were excluded from measurements because even small punctures of the body caused shrinkage. Finally, parasite sex was recorded when possible (i.e., when morphological features were visible) and the presence of egg sacs was noted.

### 2.5 Data structure

The experiments for both life cycle stages were designed to ensure that the observed treatment effects truly occurred due to the temperature treatments and not due to other factors. Therefore both experiments used two separate water baths with individually regulated temperatures for each temperature level per experiment (the free‐living larval stage experiment was done in two runs resulting in four temperature baths at each temperature in total [Figure 1 and Figure S1]). These procedural measures resulted in a nested structure of our experimental design where the observations were not completely independent.

### 2.6 Statistical approach

The nested design was taken into account in the analysis by adding random effects into generalised linear mixed effect models (GLMM). However, as the data did not allow several random effects per model only the most relevant one was chosen for all final models based on model fits by Akaike's Information Criterion (AIC) values. All initial GLMM models for our three transition success outcomes (TR1–TR3) and copepodite development (DS1) and lifespan times (Figure 1) included an interaction term between the fixed effects of temperature and species to estimate species differences and species‐specific temperature differences. The final models were chosen based on model fit using AIC values by comparing the initial model with a simpler model without interaction. When the model with interaction failed to converge, the effect of temperature on the success measure was explored for the two parasite species separately to enable evaluating the temperature responses within each species. For the sake of consistency, the same was done also when a model with interaction had a poorer fit than a model without interaction. Bonferroni correction was not applied because we simplified models and did not perform multiple tests on the same data.

The data was analysed with R version 2022.07.2 + 576 (R Core Team, 2022). Data wrangling was done with packages dplyr (Wickham et al., 2023), reshape2 (Wickham, 2007) and lubridate (Grolemund & Wickham, 2011). Data visualisation was done with package ggplot2 version 3.4.0 (Wickham, 2016) combined with ggpubr version 0.5.0 (Kassambara, 2022); generalised linear mixed models with binomial responses were fitted with package lme4 version 1.1‐31 (Bates et al., 2015) and models with linear responses were further explored with package lmerTest version 3.1‐3 (Kuznetsova et al., 2017).

#### 2.6.1 Egg development success

The development success (from egg sac sections, TR1) in each temperature treatment was determined as whether or not any parasites from an egg sac section managed to hatch and develop into copepodite 1 (the infectious stage). The effect of season was first explored for both species separately with Fisher's exact tests because the distribution of egg sacs of the two species was not even across the two seasons and our limited number of observations prevented including it as a variable in full models (in which it was likely to increase variance). The other factors affecting development success were estimated with a GLMM with adaptive Gauss‐Hermite approximation using two integer scalar points, in which the success was a binomial outcome, parasite species and temperature were fixed effects and egg sac a random effect.

#### 2.6.2 Development and lifespan of copepodites

The response times for the outcomes (first hatching, first copepodite 1 [the infectious stage, DS1], 50% mortality and copepodite lifespan [which was the time from first copepodite to 50% mortality]) were analysed with GLMMs with a Poisson response, and parasite species and temperature as fixed effects and egg sac as a random effect.

#### 2.6.3 Success measures of parasite stages inside mussels

The host entry success (of infectious stages, TR2) was estimated with a GLMM using individuals not being found in the incubation water after 24 h as the binomial response, with temperature and parasite species as fixed effects and host as a random effect. This resulted in *N* = 2400 for host entry success. The infection success (of parasitic stages, TR3), which means that the parasite individual was found in the host intestine still at dissection showing that it did not only manage to enter the host but also infected it, was estimated with a similar binomial GLMM. This model used only the parasites that had success at the previous transition step (TR2 host entry success). To prevent the appearance of second‐generation larvae interfering with the estimates of the infection success, we excluded juvenile parasites (<2 mm based on Goedknegt, Thieltges, et al., 2018) from the infection success population after at least one female had grown past the minimum size for egg production in that temperature, either in that or in any previous dissection. This resulted in the *N* = 1911 for infection success.

#### 2.6.4 Growth and development times of parasites inside mussels

The size of parasites over time, by species and temperature was plotted to explore the possible impact of temperature. The effect of temperature, parasite species and time since entering the host on egg‐bearing female parasite length was explored with a GLMM with a gaussian response and temperature, parasite species and time as fixed effects, and the host as a random effect. The effect of temperature and time on female reproductive size was then explored for both species separately because the limited data did not allow for adding interactions between the fixed effects in the main model and we wanted to know if there were species‐specific temperature effects. The time to reach the reproductive female size (DS2) at each temperature was estimated for both species with linear regression fitted with logarithmically transformed parasite length data. Only observations until the first detection of females of reproductive size were included to limit the estimate to the fast growth phase before resource allocation for reproduction. This resulted in *N* = 153 for parasite growth (103 of *M. intestinalis* and 50 of *M. orientalis*). 53 parasites came from 10°C, 53 from 14°C, 17 from 18°C, 19 from 22°C and 11 from 26°C treatments. The observations in the highest temperature were less numerous and clustered into fewer hosts than in the other temperatures partially because of high host mortality at 26°C (18/30, whereas other temperatures had only two or less mussel deaths each). The models were forced to cross the y‐axis on day 0 at the length of an infectious copepodite 1. This length (intercept) was the midpoint of a range given in the literature; 0.47 mm (0.4–0.54 mm) for *M. intestinalis* and 0.27 mm (0.24–0.29 mm) for *M. orientalis* (Gee & Davey, 1986; Goedknegt, Bedolfe, et al., 2018). In temperature treatments where the reproductive size was already reached by the first dissection, fitting regression lines based on only two time points (days 0 and 56) was not sensible and thus the time for reaching the reproductive size in those categories was estimated as <56 days.

### 2.7 Parasite life cycle duration and number of parasite life cycles per year

The time required to complete the whole life cycle at each temperature was estimated for both species by adding up the development times of both life stages, i.e. the mean time for the first parasites of an egg sac section to reach the infectious copepodite 1 stage (DS1) and the time from the infectious copepodite 1 entering a host to the appearance of the reproductive size female parasites (DS2), which were estimated with the linear regression models mentioned above.

This information on life cycle duration at each temperature was then used to estimate the number and timing of life cycles in past, present and future water temperatures of the Wadden Sea. Each experimental temperature was used as the midpoint of a 4°C temperature window (10°C for <12°C window, 14°C for 12–16°C window, 18°C for 16–20°C window, 22°C for 20–24°C window, 26°C for >24°C window). As temperature data we used daily water temperature measurements taken on the Wadden Sea side of the island of Texel by the Royal Netherlands Institute for Sea Research from 1981 to 2021 (08:00 AM values, UTC). We calculated the mean temperature for each day of a year by decade (each the mean of 10 measurements), with this we calculated the number of days with water temperature at each temperature window on a mean temperature year of each decade. This was then used to calculate the number of life cycles both parasites could have completed in an average year of each decade: the number of days at a temperature window divided by the number of days needed to complete the life cycle in the window resulting in the number of life cycles completed at each temperature, which was then added up to get the total number of life cycles per average year. Our future predictions were based on the Intergovernmental Panel on Climate Change (IPCC) predictions for scenarios SSP1‐1.9, SSP2‐4.5, SSP5‐8.5 with the respective global temperature increases of 1.5°C, 2.7°C and 4.4°C from the baseline (Pörtner et al., 2022). We used 1982–1991 as the baseline for the predictions because that was the first decade for which continuous daily observations were available and because based on earlier lower resolution data the water temperature of the Wadden Sea did not have an increasing trend before the 1980s.

## 3 RESULTS

### 3.1 Free‐living larval stages

We collected 21 egg sacs of *M. intestinalis* and 27 egg sacs of *M. orientalis*, resulting in a total number of 48 egg sacs (24 per season), cut into 240 egg sac sections. Parasite egg availability was poor for *M. orientalis* in the autumn and for *M. intestinalis* in the spring, resulting in an uneven distribution of parasites on different rounds (18/21 of *M. intestinalis* eggs and 6/27 of *M. orientalis* eggs were incubated on the autumn run). The ripeness of egg sacs was similar across the two species at the start of the experiment: 5/21 of *M. intestinalis* eggs and 8/27 of *M. orientalis* eggs had embryonic eyes on day 0.

#### 3.1.1 Development success from egg sacs

The proportion of egg sac sections from which parasites hatched and developed into infectious stages across the five temperatures was 62%–81% for *M. intestinalis* and 70%–74% for *M. orientalis*. The egg development success of each parasite was higher in the season when egg availability was better (Fisher's exact tests for both species with *p*‐value < .001). All three egg sacs of *M. intestinalis* collected in the spring failed to hatch at all five temperatures (15/15), whereas in the autumn only 10/90 (11%) egg sac sections failed. For *M. orientalis* 18/30 (60%) of egg sac sections failed in the autumn, whereas in the spring only 19/105 (18%) of them failed. Furthermore, 10/12 of the successful *M. orientalis* autumn observations originated from two egg sacs that were successful at all temperatures and two observations of one egg sac that was successful only at 10°C and 18°C. Parasite species and temperature did not affect development success (TR1) in the full model with both species, and including an interaction term between the fixed effects did not improve model fit (Table 1). In the parasite‐specific models the development success of *M. intestinalis* was lower at 26°C compared to the intercept at 10°C (odds ratio with 95% confidence intervals: 0.0 (0.0–0.7), Table 1).

**TABLE 1:** Binomial GLMMs investigating the effect of temperature and parasite species on success outcomes for transitions (TR1–TR3) from one life stage to another.

	TR1 development success			TR2 host entry success			TR3 infection success		
	Full model	*Mytilicola intestinalis*	*Mytilicola orientalis*	Full model	*Mytilicola intestinalis*	*Mytilicola orientalis*	Full model	*Mytilicola intestinalis*	*Mytilicola orientalis*
Intercept	41.9 (2.4–745.0)*	419.5 (2.8–63977.6)*	20.3 (1.0–381.8)*	78.6 (27.5–225.3)***	52.3 (17.4–157.0)***	240.9 (34.2–1698.3)***	0.3 (0.2–0.6)***	0.3 (0.2–0.5)***	0.1 (0.0–0.3)***
Species (*M. orientalis* vs. *M. intestinalis*)	0.9 (0.0–25.3)	NA	NA	1.7 (0.8–3.7)	NA	NA	0.3 (0.2–0.5)***	NA	NA
14°C	1.6 (0.2–10.3)	1.0 (0.0–51.4)	2.3 (0.2–28.4)	0.6 (0.2–2.0)	0.5 (0.1–2.1)	0.8 (0.1–8.4)	1.3 (0.6–2.9)	1.6 (0.7–3.7)	0.9 (0.2–3.7)
18°C	1.6 (0.2–10.3)	1.0 (0.0–51.4)	2.3 (0.2–28.4)	0.6 (0.2–2.1)	0.9 (0.2–3.8)	0.4 (0.0–4.0)	1.4 (0.6–3.1)	2.2 (1.0–4.8)*	0.8 (0.2–3.3)
22°C	0.6 (0.1–4.0)	0.2 (0.0–8.8)	1 (0.1–13.2)	0.4 (0.1–1.4)	0.4 (0.1–1.6)	0.4 (0.0–4.0)	1.6 (0.7–3.3)	2.4 (1.1–5.1)*	0.8 (0.2–3.4)
26°C	0.3 (0.0–1.8)	0.0 (0.0–0.7)*	2.3 (0.2–28.4)	0.3 (0.1–0.9)*	0.5 (0.1–1.8)	0.1 (0.0–1.2)	2.5 (0.9–6.7)	1.3 (0.5–3.8)	5.1 (0.9–30.4)

*Note*: With successful development of an egg sac section, parasites hatch and develop into the first infectious copepodite. With successful host entry, an infectious copepodite manages to be ingested by a mussel in 24 h. With successful infection, a parasite that was first successful in host entry manages to stay in the host until the end of the experiment (see Figure 1). Odds ratios with 95% confidence intervals are based on generalised linear mixed models with the egg sac (development) or host (host entry, infection) as random effects and temperature and parasite species as fixed effects, these models did not include interactions between the fixed effects. Significance levels: *p < .05, **p < .01, ***p < .001.

#### 3.1.2 Development and lifespan of copepodites

**FIGURE 2:**
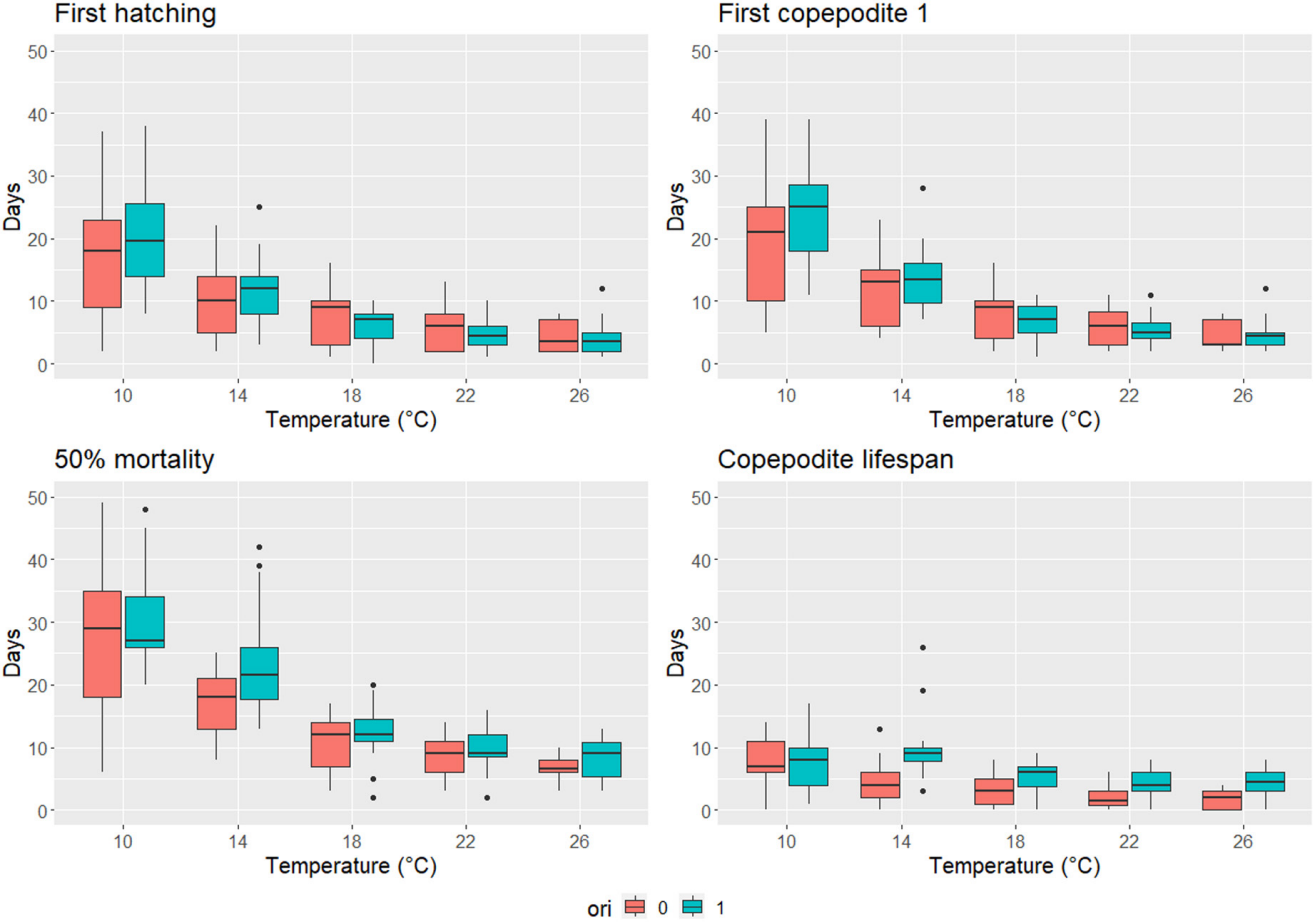
Duration of the free‐living stages of both parasite species along a temperature gradient (“response times”). Results for *Mytilicola intestinalis* are shown with orange and for *Mytilicola orientalis* with turquoise. Boxes show the median (line), 75% quantiles (box) and 90% quantiles (whiskers). First hatching, first copepodite 1 (the infectious stage) and 50% mortality show the number of days from the beginning of egg incubation and the copepodite lifespan is the time from the appearance of the first copepodite to 50% mortality. All mean times with 95% confidence intervals are available in Table S2. See Table 2 for the outputs of generalised linear mixed effect models that estimate the effects of temperature and species on these outcomes.

The time to first hatching, reaching the first copepodite 1 stages (the infectious stage, DS1), 50% mortality and the copepodite lifespan were all significantly shortened by increasing temperature in the whole range of 10–26°C with the exception of copepodite lifespan in *M. orientalis* that was non‐significantly increased at 14°C compared to the intercept at 10°C (Table 2, Figure 2 and Tables S1 and S2). *Mytilicola orientalis* reached the first copepodite 1 stage slower at 10°C but at temperatures 18–26°C the effect of species disappeared through a significant negative interaction between species and temperature, and a similar effect was seen also for first hatching (Table 2). The lifespan of *M. orientalis* copepodites was longer than those of *M. intestinalis* at 14°C, 22°C and 26°C (Table 2). For 50% mortality the model without interaction between temperature and parasite species had a better fit than the model with interaction. The model with interaction did not find significant interactions and in the model without interaction it took longer for *M. orientalis* than *M. intestinalis* to reach 50% mortality across temperatures (Table 2). The species‐specific 50% mortality models for *M. intestinalis*, and *M. orientalis* confirmed that the temperature effect was similar for both species across the range of temperatures (Table S1). Free‐living stages of both parasite species appeared to move less in 10–14°C than in higher temperatures.

**TABLE 2:** The impact of temperature and parasite species on free‐living larval stage response times.

	First hatching				First copepodite (DS1)				50% mortality				Copepodite lifespan			
	Estimate	Std. error	*z*‐Value	*p*‐Value	Estimate	Std. error	*z*‐Value	*p*‐Value	Estimate	Std. error	*z*‐Value	*p*‐Value	Estimate	Std. error	*z*‐Value	*p*‐Value
Intercept (*Mytilicola intestinalis*, 10°C)	2.712	0.145	18.742	<.001***	2.872	0.122	23.511	<.001***	3.247 (3.254)	0.088 (0.091)	37.044 (35.707)	<.001***	1.949	0.123	15.848	<.001***
14°C	−0.554	0.094	−5.899	<.001***	−0.529	0.088	−5.993	<.001***	−0.423 (−0.500)	0.050 (0.075)	−8.498 (−6.681)	<.001***	−0.425	0.140	−3.033	.002**
18°C	−0.918	0.106	−8.644	<.001***	−0.947	0.102	−9.311	<.001***	−0.954 (−0.927)	0.061 (0.086)	−15.577 (−10.730)	<.001***	−0.874	0.163	−5.377	<.001***
22°C	−1.145	0.116	−9.918	<.001***	−1.147	0.115	−9.950	<.001***	−1.204 (−1.183)	0.064 (0.095)	−18.751 (−12.453)	<.001***	−1.301	0.194	−6.692	<.001***
26°C	−1.189	0.140	−8.490	<.001***	−1.200	0.140	−8.545	<.001***	−1.359 (−1.290)	0.073 (0.113)	−18.725 (−11.411)	<.001***	−1.430	0.223	−6.425	<.001***
Species (*Mytilicola orientalis*)	0.337	0.189	1.777	.076	0.348	0.163	2.138	.033*	0.226 (0.210)	0.113 (0.124)	2.001 (1.693)	.045* (.090)	0.077	0.170	0.453	.651
14°C*Species	−0.146	0.127	−1.148	.251	−0.125	0.119	−1.051	.294	NA (0.134)	NA (0.101)	NA (1.329)	NA (.184)	0.610	0.182	3.355	<.001***
18°C*Species	−0.377	0.147	−2.562	.010*	−0.289	0.140	−2.069	.039*	NA (−0.052)	NA (0.122)	NA (−0.422)	NA (.673)	0.425	0.217	1.956	.051
22°C*Species	−0.469	0.164	−2.857	.004**	−0.388	0.159	−2.439	.015*	NA (−0.034)	NA (0.129)	NA (−0.265)	NA (.791)	0.678	0.241	2.816	.005**
26°C*Species	−0.648	0.189	−3.424	<.001***	−0.562	0.184	−3.049	.002**	NA (−0.108)	NA (0.148)	NA (−0.729)	NA (.466)	0.762	0.266	2.866	.004**

*Note*: Outputs from generalised linear mixed effect models with Poisson response and egg sac as a random effect. For 50% mortality the model without interaction between fixed effects had a better fit than the model with interaction, the results for the poorer model in parentheses. Significance levels: *p < .05, **p < .01, ***p < .001.

Abbreviation: Std. error, standard error.

### 3.2 Stages inside mussels

#### 3.2.1 Success measures of parasite stages inside mussels

For the host entry success (TR2), the initial model with interaction term between the fixed effects of parasite species and temperature failed to converge. In a model without interaction between the fixed effects, parasite species did not have an effect and only the highest temperature (26°C) had a significant negative impact on the ability of parasite larvae to enter hosts (Table 1). When explored for the two parasite species separately, the negative effect of 26°C on host entry success was lost (Table 1). For the infection success (TR3), the initial model with interaction term between the fixed effects of parasite species and temperature failed to converge. In a model without interaction between the fixed effects, *M. orientalis* had a significantly poorer infection success than *M. intestinalis* (odds ratio with 95% confidence intervals for *M. orientalis* over *M. intestinalis*: 0.3 (0.2–0.5) but temperature did not have an effect [Table 1]). When explored for the two parasite species separately, 18°C and 22°C had significant positive effects on infection success in *M. intestinalis* but not in *M. orientalis* (Table 1). The stages inside mussels of both parasite species appeared paler in 22°C and 26°C than in lower temperatures.

#### 3.2.2 Growth and development times of parasites inside mussels

**FIGURE 3:**
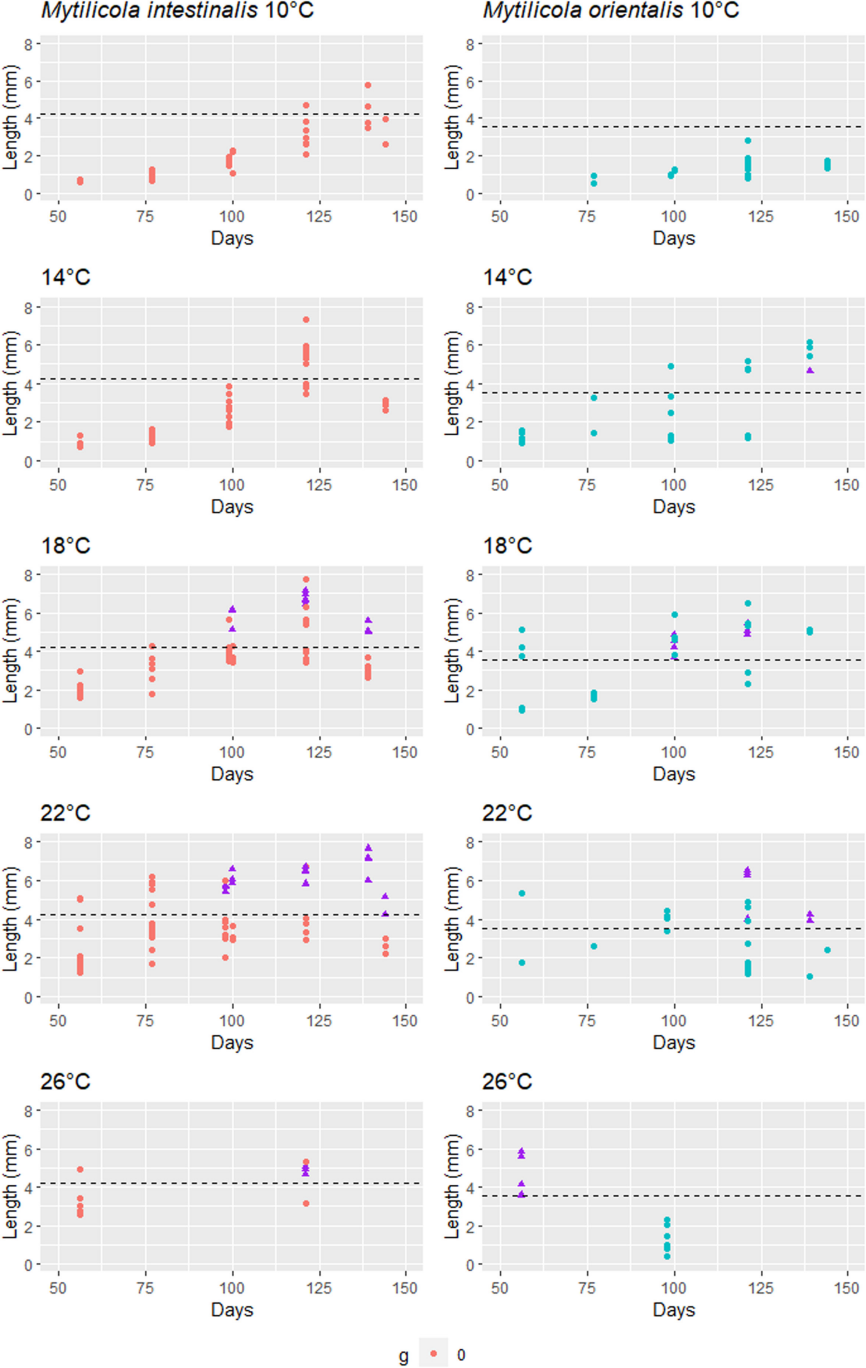
Effect of temperature on the growth of the two parasite species inside the mussels. Results for *Mytilicola intestinalis* are shown on the left side and for *Mytilicola orientalis* on the right side. Horizontal lines represent the minimum reproductive size of females and purple triangles show females with egg sacs. The *x*‐axis gives the time from the day of entering the host.

Based on parasite egg detection, reproduction had clearly started during the experiment at temperatures between 18–26°C for *M. intestinalis* and 14–26°C for *M. orientalis* (purple triangles in Figure 3). The total number of females with egg sacs was 36 for *M. intestinalis* and 31 for *M. orientalis*. The length of the shortest egg‐bearing female was 4.23 mm in *M. intestinalis* and 3.55 mm in *M. orientalis*, and these were used as our cut‐off values for reproductive size. Temperature and time did not have significant effects on the size of egg‐bearing females (see Figure S2a,b for size distribution across temperatures and Table S3 for model output). *Mytilicola intestinalis* egg‐bearing females appeared smaller at 26°C than at other temperatures (Figure S2a,b) but because of the small number of observations at 26°C and the clustering of observations in hosts, the finding was not statistically significant (Table S3). Based on parasite lengths by temperature, species and time (Figure 3), *M. intestinalis* had already reached reproductive size at 22°C and 26°C and *M. orientalis* at all temperatures above 14°C (18–26°C) by the time of our first dissection at 56 days. All regression models fitted to logarithmically transformed parasite length data of the other temperature treatments had good fits (*R* ^2^: .905–.976, *p* < .001, see the regression lines in Figure S3) and the time for reaching the reproductive size in those categories was estimated from them (DS2, Table 3).

**TABLE 3:** Mean life cycle durations (days) of the two parasite species in different temperature regimes.

	DS1 free‐living		DS2 inside mussels		The full duration	
	*Mytilicola intestinalis*	*Mytilicola orientalis*	*Mytilicola intestinalis*	*Mytilicola orientalis*	*Mytilicola intestinalis*	*Mytilicola orientalis*
10°C	20 (14–26)	24 (21–28)	174	198	194	222
14°C	12 (9–15)	14 (11–16)	129	115	141	129
18°C	8 (6–10)	7 (6–8)	88	<56	96	<63
22°C	6 (4–8)	5 (4–7)	<56	<56	<62	<61
26°C	5 (3–6)	5 (3–6)	<56	<56	<61	<61

*Note*: The time to complete the free‐living stage was the mean time for an egg sac to ripen, hatch and go through two larval stages to produce the first infectious copepodites, 95% confidence intervals are shown in parentheses. The time inside mussels was the minimum time it took for an infectious stage to grow into the smallest size for female reproduction after entering a host in the infection experiment. In the highest temperatures that reproductive size was already reached by the first dissection at day 56 and in the lower temperatures the time was estimated from linear regression models fitted into logarithmically transformed length data (see Figure 3 for parasite sizes and Figure S3 for regression line fits).

### 3.3 Parasite life cycle duration and number of parasite life cycles per year

Both parasites developed faster at higher temperatures (DS1 + DS2, Table 3). The main observed difference between the two species in this range of temperatures was that *M. orientalis* was slower only at 10°C and it reached the maximum development speed already by 18°C, whereas *M. intestinalis* reached it by 22°C (Table 3, Figures 2 and 3). Because the effect of temperature on development speed was the same in the two highest temperatures, they were merged into one temperature window for assessing the effect of climate change for parasite development (Tables 3 and 4).

**TABLE 4:** The number and timing of life cycles of two closely related parasitic copepods in different water temperature scenarios for the southern Wadden Sea.

Life cycles completed per year												
Temperature window	1982–1991			2012–2021			SSP2‐4.5 increase by 2100 from 1982 to 1991			SSP5‐8.5 increase by 2100 from 1982 to 1991		
	Days	*Mytilicola intestinalis*	*Mytilicola orientalis*	Days	*Mytilicola intestinalis*	*Mytilicola orientalis*	Days	*Mytilicola intestinalis*	*Mytilicola orientalis*	Days	*Mytilicola intestinalis*	*Mytilicola orientalis*
<12°C	206	1.06	0.93	190	0.98	0.86	162	0.84	0.73	145	0.75	0.65
12–16°C	83	0.59	0.64	60	0.43	0.47	58	0.41	0.45	58	0.41	0.45
16–20°C	76	0.79	1.23	101	1.05	1.63	87	0.91	1.40	78	0.81	1.25
>20°C	0	0	0	14	0.23	0.23	58	0.95	0.96	84	1.38	1.41
Total	365	2.44	2.8	365	2.69	3.19	365	3.10	3.55	365	3.35	3.76

*Note*: The number of days at each temperature window was calculated from the mean decadal 8 am temperature for each day. SSP2‐4.5 and SSP5‐8.5 predictions were estimated from the baseline (1982–1991) and they predict 2.7°C and 4.4°C mean increase by 2100 respectively.

**FIGURE 4:**
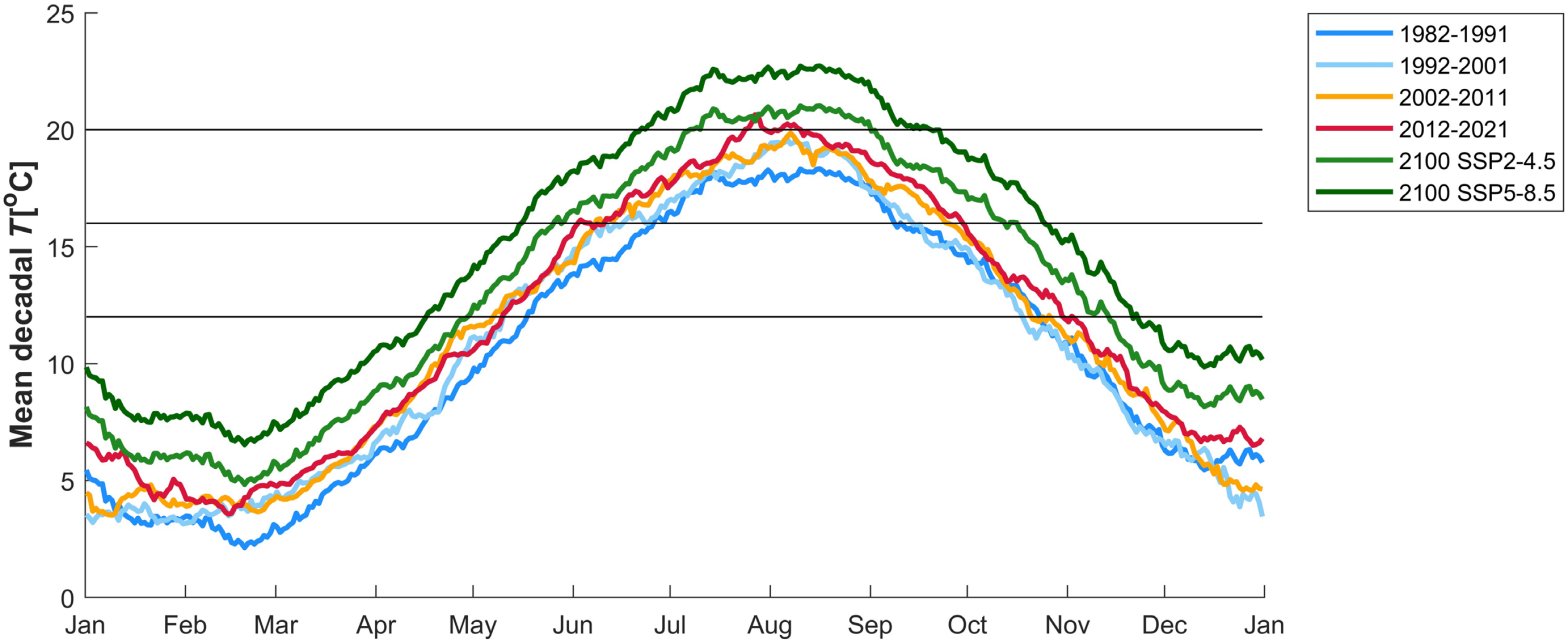
The past, present and predicted future water temperature of the ecosystem from which parasite specimens were sourced (the southern Wadden Sea) measured at the NIOZ jetty (53.002°N, 4.789°E) on the island of Texel. The lines represent decadal mean temperatures for the selected 10‐year periods between 1982 and 2021 and the SSP2‐4.5 mid‐range and SSP5‐8.5 top‐range predictions for 2100. SSP2‐4.5 and SSP5‐8.5 predictions were estimated from the baseline (1982–1991) by adding the predicted 2.7°C and 4.4°C mean increase respectively. SSP1‐1.9 prediction of a mean increase of 1.5°C from the baseline was not included because the 2012–2021 observational mean is already close to that level. The horizontal lines represent the temperature windows used to categorise and predict life stage durations (Table 4).

The temperature increase in the southern Wadden Sea from 1982–1991 to 2012–2021 was on the level of the SSP1‐1.9 low range prediction of a mean increase of 1.5°C and has occurred evenly across the year (red versus dark blue line in Figure 4). Thus, only the higher predictions of SSP2‐4.5 (mid‐range) and SSP5‐8.5 (top‐range) for 2100 were added to the observed data (green lines in Figure 4). Using 1982–1991 as the baseline before clear climate change effects, days in the fastest development speed window for *M. intestinalis* (>20°C) did not use to occur on an average year (Figure 4), whereas they may increase from the current 14 up to 84 by 2100 (Table 4 and Figure 4). Since 1982–1991, the number of days in the temperature windows with fast parasite life cycles for both parasites (>16°C) has already increased from 76 to 115, and it may increase up to 162 in the highest temperature prediction for 2100 (Tables 4 and Figure 4). These changes result in more life cycles completed on an average year with the increase taking place during the warmest months (Figure 4 and Table 4).

## 4 DISCUSSION

Our experiments revealed that an increase in temperature increases the number of life cycles both parasite species can complete within a year, with faster life cycles being most pronounced during the warmest season. This can result in a high infection pressure on hosts in times when they are also the most exposed to direct temperature stress. In contrast to our expectation, the acceleration of life cycles with higher summer temperatures (from 18°C to 22°C) was slightly stronger for the established species than for the more recent invader that appears to benefit more from the temperature increase at the lower end (from 10°C to 14°C) of this temperature range. The impact of temperature on all three success measures that were used as estimates for transitions between life cycle stages was weak within the current and predicted temperature ranges for the Wadden Sea. Thus, the main effect of temperature on these two parasite species appears to be via development speed and not life cycle transition success. Combining all outcomes of this study, the established invader *M. intestinalis* is better adapted for the low range of current temperatures (around 10°C) and a temperature above the currently predicted range (to 26°C) would push it past its adapted range, whereas the more recent invader *M. orientalis* barely develops in the lowest current temperatures but can cope with extreme temperatures (around 26°C) that may occur during heat waves in this ecosystem.

### 4.1 Success measures

In other parasites, high temperature can increase the infectivity of the free‐living stages, and also the infection success of the parasitic stages through a decrease in host immune responses (Barber et al., 2016). However, in our experiments, temperature effects on all three success measures for life cycle transitions were relatively small across all temperatures within the predicted range for the Wadden Sea (<26°C by 2100, Figure 4). The development success of eggs (TR1) of *M. intestinalis* decreased at 26°C, but it was not affected in *M. orientalis*. Seasonal limitations in egg availability precluded estimating the impact of egg development temperature on the development success, but the very limited overlapping observations point to both parasites possibly having a lower success in their “low” season, and this potential effect needs to be investigated in the future. Also, the host entry success of copepodites (TR2) was uniform in the range 10–22°C for both parasites, whereas our main model combining both species found lower entry success at 26°C. This uniformity of entry success was surprising because we expected that temperature‐associated differences in activity levels should affect the ability of copepodites to swim towards their hosts, as well as because the filtration rate of blue mussels is affected by temperature (Vajedsamiei et al., 2021). The slightly lower entry success at 26°C may have been because of the faster development at the highest temperature and thus passing through the optimal stage for infection before entering the host. The evidence for this was our observation of many of the remaining copepodites in the highest temperature having already grown an extra pair of limbs as a sign of reaching the next stage (copepodite 2, see Gee and Davey (1986) for a detailed explanation about life cycle stages), along with the shorter copepodite lifespans at the highest temperatures of our free‐living stage experiment. Another likely explanation is the drop in blue mussel filtration rates between 22°C and 26°C (Vajedsamiei et al., 2021), which makes entering the gastrointestinal tracts of mussels less likely. The experimental design that prioritised using infectious copepodites from the same egg sacs for all temperature treatments of an infection batch did not allow for parasite acclimatisation, because eggs hatch at different speeds at different temperatures and all treatments of a batch were started on the same day to minimise biases from unobserved factors. This may have led to a cold shock for the copepodites in the lowest temperatures. The uniformity of the entry success, however, suggests that infectious stages of both parasite species are not strongly affected by cold temperatures.

In general, the infection success (TR3, the ability of parasites that entered hosts to infect them) was better for *M. intestinalis* than *M. orientalis*, which has been shown before by Feis et al. (2022). One possible reason for the difference between species could be the longer time of *M. intestinalis* to adapt to the host and environment compared to *M. orientalis* (~90 vs. ~20 years). Another, possibly more convincing explanation is the former being a specific blue mussel parasite, whereas the latter is a generalist capable of infecting several host species. *Mytilicola orientalist* has generalist tendencies also in the native range in Japan, where it infects invasive Mediterranean mussels (*Mytilus galloprovincialis*) along with Pacific oysters (Feis et al., 2019). The infection success of *M. intestinalis* was increased at 18°C and 22°C, but not at 26°C. This may indicate the highest temperature being past the temperature optimum of *M. intestinalis* or result from the highest temperature compromising the filtration by the hosts (Vajedsamiei et al., 2021), indirectly affecting the food availability of parasites in a long‐term experiment like this. Our infection success was not an exact measure of the original parasites staying in the hosts until the time of dissection because of the unexpected appearance of the next generation in the highest temperature treatments, and the inability to exclude all offspring from the data. Our result should therefore be interpreted as an integrated outcome that included some of the positive effects of several life cycles in the higher temperatures. One factor that may have added noise to our infection success observations is natural variation in mussel susceptibility to infection (Levakin et al., 2013), but this effect was randomly distributed over treatment groups.

### 4.2 Development speed

In contrast to life cycle transition success (TR1–TR3), temperature did have a pronounced effect on development speed (DS1–DS2). The free‐living stages of both parasites developed faster (DS1) in higher temperatures. In a natural setting, this effect decreases the time they are at risk for being predated but also the potential time to find a host due to a shorter lifespan. On the other hand, the increase in temperature may also increase the predation pressure per unit of time due to an increase in predator metabolic rates (Barber et al., 2016). The acceleration of free‐living larvae development was most pronounced across the lower range of our temperatures (10–18°C), whereas differences between temperature categories in the higher range (18–26°C) were almost non‐existent. This indicates that the free‐living stages of both parasites benefit more from milder winters than hotter summers. This, combined with the decrease in development success (TR1) of *M. intestinalis* and host entry success (TR2) of both species at 26°C, suggests that this extreme temperature is outside the adapted range at least for *M. intestinalis*.

The temperature effect on the development speed of the life cycle stages inside mussels (DS2) was more even across temperatures for *M. intestinalis* than for *M. orientalis*. For the latter, the three highest temperatures resulted in fast maturation, while there was barely any growth at the lowest temperature (10°C). This may reflect adaptation to the native range of *M. orientalis*, where water temperature does not go below 13°C and temperatures >20°C are more common than in the Wadden Sea (Japan Meteorological Agency, 2023). The origin of *M. intestinalis* remains unclear but the parasite species has had a longer time to adapt to the environmental conditions in the Wadden Sea ecosystem. The mean annual water temperature in the Wadden Sea is still <13°C but it has increased from <10.5°C to approximately 12°C in the last 20 years (Royal Netherlands Institute for Sea Research, 2023), and the number of days per year with water temperature <12°C is predicted to further decrease with climate change (Table 4).

All three egg‐bearing *M. intestinalis* females observed at 26°C were smaller than in the lower temperatures (Figure S2) but that observation was not statistically significant due to the clustering of these individuals in a single mussel. More work needs to be done on the possible temperature impact on reproductive size. This work should also include the possible temperature impact on the number of eggs produced per female both directly and indirectly through female size, and the impact of egg development temperature on their development success. According to Barber et al. (2016), the development temperature of eggs may also affect the lifetime reproductive output of the parasites through carry‐over effects across life stages.

### 4.3 Lifespan

Increasing temperature was expected to shorten the lifespan of both life cycle stages because temperature‐driven increases in metabolic rate shorten the lifespan of ectothermic animals (Gillooly et al., 2001). The effect is especially prominent for non‐feeding free‐living stages of aquatic parasites such as trematodes, and also the free‐living stages of the two parasitic copepods of this study, that deplete their energy resources faster at higher temperatures (Barber et al., 2016). Our impression of the free‐living stages being mostly immobile at 10–14°C while being very active at higher temperatures, and the fact that copepodite life spans decreased with temperature in the experiments support that. Interestingly, for *M. intestinalis* the effect of temperature on the lifespan of infectious copepodites was close to linear across our range of temperatures (Figure 2), whereas *M. orientalis* copepodite 1 lifespan did not decrease from 10°C to 14°C (a non‐significant increase), and it had longer lifespans than *M. intestinalis* at all temperatures above 10°C (the difference being only almost significant at 18°C with *p* = .051, Table 2). We used the time from reaching the copepodite 1 stage to 50% mortality as a proxy for copepodite 1 lifespan. Although it may not perfectly describe the time the parasite is infectious, it provides the best available estimate. The exact duration each copepodite stays in the infectious stage is difficult to determine because it is a combination of developing past the particular life stage (Gee & Davey, 1986) and losing the ability to swim linearly (Eike Petersen, personal communication), the evaluation of the latter being further complicated by the difference in activity levels by temperature.

The lifespan of the stages inside mussels appeared to differ by temperature in both species (Figure 3). The appearance of the second generation limited our ability to estimate lifespan, since especially later in the experiment it was not possible to determine which parasite belonged to which generation. For *M. intestinalis*, lifespan has been studied before by investigating climatic gradients along their European range: in Galicia (Spain) the lifespan has been suggested to be below 3 months, whereas in the Netherlands in the 1960s it was estimated to be 9–10 months (Korringa, 1968). Our results show the importance of local temperature conditions for these differences: The first generation of *M. intestinalis* appeared to die out by 11 weeks at 26°C, between 17 and 20 weeks at 14–18°C and after 20 weeks at 10°C (Figure 3). The lower number of parasite observations made this estimation more difficult for *M. orientalis*, but the impact appears mostly similar across the two species.

### 4.4 Climate change effects

The higher water temperature compared to the average in the 1980s has already increased the potential number of annual life cycles of both parasites, and this trend is predicted to continue. Surprisingly, the increase in summer temperatures appears to benefit the established species more than the recent invader (faster growth from 18°C to 22°C and an increase in infection success at 18–22°C in *M. intestinalis*). If the predicted increase were larger so that it would result in hitting the upper temperature limit of *M. intestinalis* around 26°C, the decrease in egg development success might compensate for this effect. Thus, our expectation that the thermal range of the recent invader from a warm climate would be more suitable for the increasing temperatures due to climate change was only partially met. Interestingly, the water temperature increase expected for the Wadden Sea does not appear to be high enough to hit the upper limits of the established invader. This is interesting considering the observed decline in *M. intestinalis* prevalence on the island of Sylt on northern Wadden Sea 2013–2019, which has been suggested to be related to the arrival of *M. orientalis* (Feis et al., 2022). Based on our results, the increase in water temperature is not likely to contribute to that decline. Because the development speed of the recent invader appeared to decline steeply already with a temperature decrease from 14°C to 10°C it would be interesting to explore the differences in winter survival of these two species when temperatures are <10°C and can reach <3°C for intertidal mussels exposed during the low tide. How the projected increase in temperatures at both ends of the temperature spectrum will influence survival in the future (Figure 4) can be crucial for predicting differential population growth of both species. Our estimates of the effect of temperature in this range on the number of parasite life cycles per year is rather conservative because we assumed a uniform impact at temperatures <12°C and >20°C to err on the side of caution in the absence of data. Also based on the already occurred Wadden Sea warming in just 40 years the SSP2‐4.5 mid‐range prediction for 2100 appears optimistic. So far, the increase in Wadden Sea water temperature has been close to uniform across the seasons with a slightly higher increase during the summer (see Figure 4), therefore our predictions with uniform temperature increase appear realistic.

Climate change is predicted to increase the frequency of extreme weather events and especially intertidal mussels are already exposed to fast temperature changes during the low tide (Seuront et al., 2019). Repeated heat stress causes mussel mortality already by itself (Seuront et al., 2019). The increase in parasite life cycles completed during the warmest season and the resulting elevated infection pressure can potentially amplify stress and mortality. Copepod infections in other marine ectotherms (fish) tend to peak in the summer, possibly reflecting this type of dynamic, and parasites with simple life cycles are more likely to benefit from increasing temperature than parasites with complex life cycles because they are less impacted by phenological shifts of hosts (Marcogliese, 2001; Poulin, 2020). Elevated temperatures can increase mortality due to secondary infections in mussels when infected with *M. intestinalis* (Demann & Wegner, 2019). However, the traits involved in host parasite interactions can vary among populations within *M. intestinalis* (Feis et al., 2016, 2018) and the impact of *M. orientalis* on host immune function, growth and reproduction is not known at all. Furthermore, temperature can also affect the trophic interactions between the parasites and the hosts, and the possible effect of parasites on the temperature‐tolerance of hosts needs to be explored both in the short‐ and in the long‐term. For example, in common cockles infected with trematodes temperature is not the only predictor for seasonal trends and the optimal temperature for cercariae emergence differs by the geographical location (de Montaudouin et al., 2016). These findings necessitate further research into the impact of parasites on the acute and chronic heat‐tolerance of hosts. In the time frame until 2100, evolution is likely to modify both the temperature adaptation of the two parasite species and their interaction with mussel hosts in this relatively new host–parasite system undergoing rapid warming. Repeating this study with parasites and hosts from a more southern region of the invaded range would help in estimating the capacity for temperature adaptation and make our predictions more robust.

## 5 CONCLUSIONS

The recent invader originating from a warmer system is better adapted to cope with heat wave temperatures and benefits more from milder winters than the established invader, whereas the established invader gains more benefit from the predicted increase in summer temperatures below heat wave temperatures. The data collected here is essential to estimate the future of this system with models, where the effects on individuals are scaled up to populations to predict ecosystem development. In the scaling up, it is necessary to include other climate change mediated aspects, specifically the influence temperature can have on predation and competition both for the parasites and their hosts in future studies. Despite the multitude of factors that should be considered, the simplicity of the Mytilicola‐system renders exploration of the combined effect of climate change and parasite invasions on ecosystems feasible, forming a useful building block for a more general ecosystem model for infectious disease (Hassell et al., 2021).

## AUTHOR CONTRIBUTIONS

**Elli Rosa Emilia Jolma:** Conceptualization (equal); data curation (equal); formal analysis (equal); investigation (equal); methodology (equal); project administration (equal); visualization (equal); writing – original draft (lead); writing – review and editing (equal). **Ana Born‐Torrijos:** Investigation (equal); visualization (equal); writing – review and editing (equal). **Hans Heesterbeek:** Conceptualization (equal); funding acquisition (equal); project administration (equal); supervision (equal); writing – review and editing (equal). **Anieke van Leeuwen:** Conceptualization (equal); supervision (equal); writing – review and editing (equal). **Sonja Maria van Leeuwen:** Data curation (equal); formal analysis (equal); methodology (equal); writing – review and editing (equal). **Robert H. Twijnstra:** Investigation (equal); methodology (equal); resources (equal); visualization (equal); writing – review and editing (equal). **K. Mathias Wegner:** Conceptualization (equal); formal analysis (equal); methodology (equal); supervision (equal); writing – review and editing (equal). **David W. Thieltges:** Conceptualization (equal); funding acquisition (equal); methodology (equal); project administration (equal); supervision (equal); visualization (equal); writing – review and editing (equal).

## FUNDING INFORMATION

Financial support was provided for the contribution of Ana Born‐Torrijos by the European Union’s Horizon 2020 Research and Innovation Programme under the Marie Skłodowska‐Curie grant agreement No 101027941.

## CONFLICT OF INTEREST STATEMENT

The authors declare no conflicts of interest.

## Supporting information


Figure S1.
Figure S2.
Figure S3.
Table S1.
Table S2.
Table S3.

## Data Availability

The data that support the findings of this study are openly available in https://doi.org/10.25850/nioz/7b.b.4g.
