# Supplementary material for: Warming effects on the life cycles of two parasitic copepods with different invasion histories
Source: Ecol Evol. 2024 Jun 25;14(6):e11485. doi: 10.1002/ece3.11485 (PMC11199328; doi:10.1002/ece3.11485)
Supplement: Supplementary file 1 — Figure S1. Figure S2. Figure S3. Table S1. Table S2. Table S3. [file ECE3-14-e11485-s001.docx]

**
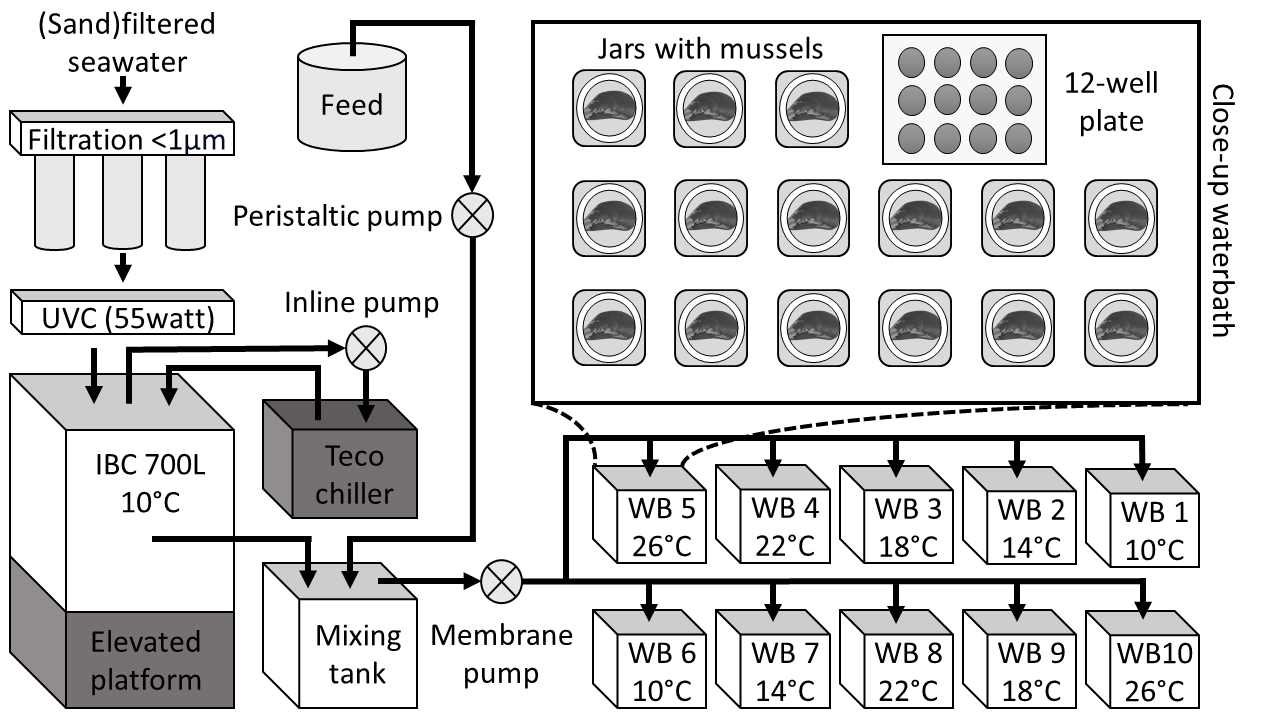
**

**Supplementary Figure 1.** The design of temperature treatments of both experiments. WB = water bath, each water bath had a separate heating and insulation system, new water entering each tank was pre-heated by pumping it through a fine tube in the water bath before entering a dripper from which it was dripped to each mussel jar. Mussel jars were used for the inside hosts experiment and the 12-well plates for the free-living larvae experiment.

**Supplementary Table 1**. The impact of temperature on free-living larval stage 50% mortality time by parasite species. Outputs from generalized linear mixed effect models with Poisson response and egg sac as a random effect. Std. error = standard error. Significance levels: *p < .05, **p < .01, ***p < .001.

|  | **50% mortality** | | | | | | | |
| --- | --- | --- | --- | --- | --- | --- | --- | --- |
|  | ***Mytilicola intestinalis*** | | | | ***Mytilicola orientalis*** | | | |
|  | **Estimate** | **Std. error** | **z-value** | **p-value** | **Estimate** | **Std. error** | **z-value** | **p-value** |
| **Intercept (10°C)** | 3.24761 | 0.106 | 30.612 | < 0.001 *** | 3.460 | 0.072 | 48.110 | < 0.001 *** |
| **14°C** | -0.50024 | 0.075 | -6.683 | < 0.001 *** | -0.35501 | 0.067 | -5.275 | < 0.001 *** |
| **18°C** | -0.92699 | 0.086 | -10.732 | < 0.001 *** | -0.97189 | 0.087 | -11.226 | < 0.001 *** |
| **22°C** | -1.1829 | 0.095 | -12.456 | < 0.001 *** | -1.20922 | 0.087 | -13.856 | < 0.001 *** |
| **26°C** | -1.28496 | 0.113 | -11.365 | < 0.001 *** | -1.38914 | 0.095 | -14.592 | < 0.001 *** |

|  | **Hatching** | | **Infectious stage** | | **50% mortality** | | **Copepodite lifespan** | |
| --- | --- | --- | --- | --- | --- | --- | --- | --- |
|  | *M. intestinalis* | *M. orientalis* | *M. intestinalis* | *M. orientalis* | *M. intestinalis* | *M. orientalis* | *M. intestinalis* | *M. orientalis* |
| **10°C** | 18 (12-24) | 21 (17-24) | 20 (14-26) | 24 (21-28) | 28 (21-34) | 30 (26-34) | 7 (5-10) | 8 (5-10) |
| **14°C** | 10 (7-14) | 12 (9-14) | 12 (9-15) | 14 (11-16) | 17 (14-20) | 23 (20-27) | 5 (3-7) | 10 (7-12) |
| **18°C** | 7 (5-10) | 6 (5-8) | 8 (6-10) | 7 (6-8) | 11 (9-13) | 12 (10-15) | 3 (2-4) | 5 (4-7) |
| **22°C** | 6 (4-8) | 5 (4-6) | 6 (4-8) | 5 (4-7) | 8 (7-10) | 10 (8-11) | 2 (1-3) | 4 (3-7) |
| **26°C** | 5 (3-6) | 4 (3-5) | 5 (3-6) | 5 (3-6) | 7 (6-8) | 8 (6-10) | 2 (1-3) | 4 (3-5) |

**Supplementary Table 2**. The mean time in days from the start of egg incubation to the first detection of each response in eggs hatched in five temperatures. 95% confidence intervals for the means are expressed in brackets.

a.


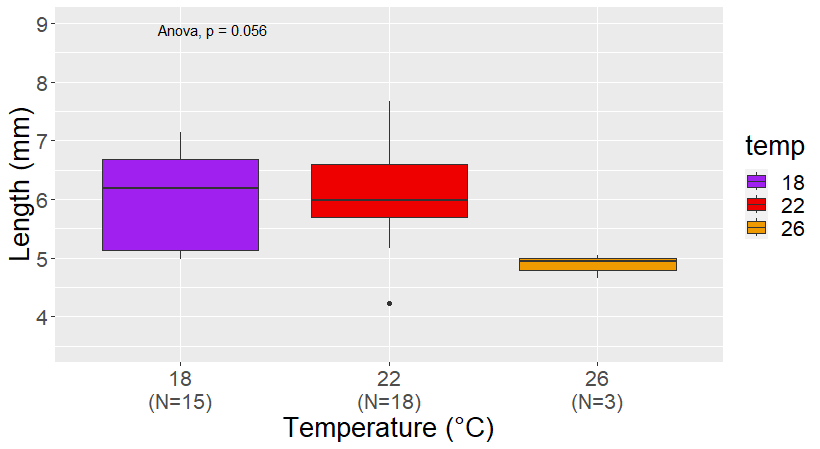


b.


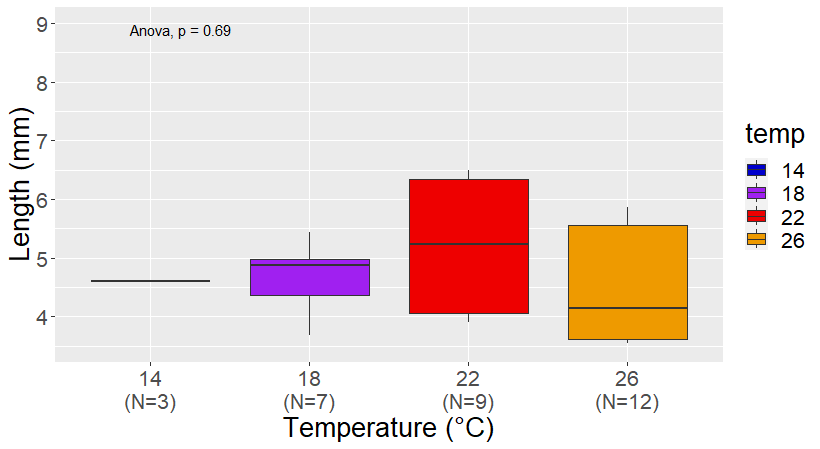


**Supplementary Figure 2.** The size of egg producing parasites by temperature for *Mytilicola* intestinalis (a) and *Mytilicola orientalis* (b).


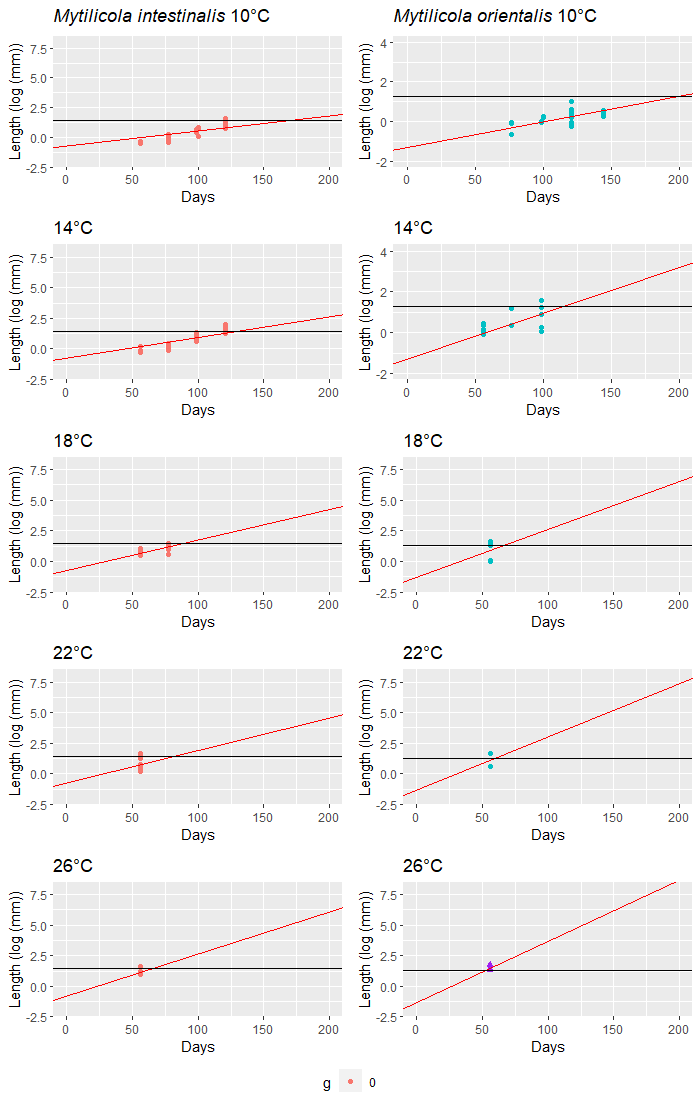


**Supplementary Figure 3**. Linear regression for parasite growth by species and temperature. Parasite length is logarithmically transformed. Only observations until the first detection of reproductive size females were included because the purpose was to estimate the impact of temperature on the fast growth phase before resource allocation for reproduction. Results for *Mytilicola intestinalis* are shown on the left side and for *Mytilicola orientalis* on the right side. Horizontal lines represent the minimum reproductive size of females and purple triangles show females with egg sacs. The x-axis gives the time from the day of entering the host. All regression lines used and intercept of 0 at day 0 from the size of an infectious copepodite (0.27 mm for *M. orientalis* and 0.47 mm for *M. intestinalis*).

**Supplementary Table 3.** The effect of temperature, species and time since entering the hosts on egg-bearing female size. The intercept of the full model was 14°C, *Mytilicola intestinalis* and day 56, the intercept of the *M. intestinalis* model was 18°C and day 99, the intercept of the *M. orientalis* model was 14°C and day 56. Std. error = standard error, Df. = degrees of freedom. Significance levels: *p < .05, **p < .01, ***p < .001.

|  | **Full model** | | | | | ***M. intestinalis*** | | | | | | ***M. orientalis*** | | | | |
| --- | --- | --- | --- | --- | --- | --- | --- | --- | --- | --- | --- | --- | --- | --- | --- | --- |
|  | **Estimate** | **Std. error** | **Df.** | **t-value** | **p-value** | **Estimate** | **Std. error** | **Df.** | **t-value** | **p-value** | **Estimate** | | **Std. error** | **Df.** | **t-value** | **p-value** |
| **Intercept** | 7.163 | 2.230 | 11.375 | 3.212 | 0.008** | 6.738 | 2.226 | 5.057 | 3.026 | 0.029* | 6.252 | | 9.354 | 2.992 | 0.668 | 0.552 |
| **Species (M.orientalis)** | -1.100 | 0.517 | 11.075 | -2.128 | 0.057 | NA | NA | NA | NA | NA | NA | | NA | NA | NA | NA |
| **Time** | -0.010 | 0.013 | 10.887 | -0.816 | 0.432 | -0.007 | 0.018 | 5.047 | -0.375 | 0.723 | -0.012 | | 0.067 | 2.987 | -0.178 | 0.870 |
| **18°C** | -0.062 | 1.193 | 12.561 | -0.052 | 0.959 | Intercept | Intercept | Intercept | Intercept | Intercept | -0.219 | | 2.561 | 3.049 | -0.085 | 0.937 |
| **22°C** | 0.095 | 1.147 | 12.887 | 0.083 | 0.935 | 0.064 | 0.665 | 4.935 | 0.097 | 0.927 | 0.079 | | 1.812 | 3.120 | 0.044 | 0.968 |
| **26°C** | -0.843 | 1.427 | 12.035 | -0.590 | 0.566 | -1.040 | 1.056 | 5.015 | -0.985 | 0.370 | -0.858 | | 5.783 | 3.000 | -0.148 | 0.891 |
